# A synthetic microbial community derived from healthy apple rhizosphere alleviates apple replant disease

**DOI:** 10.1093/hr/uhaf217

**Published:** 2025-08-25

**Authors:** Mengli Yang, Yiqi Liu, Yan Xia, Ming Li, Chuanmi Huang, Feifan Hou, Shupei Hu, Xiaoyan Zhu, Miaomiao Wang, Jiangli Shi, Ran Wan, Kunxi Zhang, Pengbo Hao, Yujie Zhao, Yu Liu, Yawen Shen, Liu Cong, Zhonghai Han, Jiancan Feng, Jian Jiao, Xianbo Zheng

**Affiliations:** College of Horticulture, Henan Agricultural University, Zhengzhou 450046, China; Institute of Horticulture, Henan Academy of Agricultural Sciences, Zhengzhou 450002, China; College of Horticulture, Henan Agricultural University, Zhengzhou 450046, China; College of Horticulture, Henan Agricultural University, Zhengzhou 450046, China; College of Horticulture, Henan Agricultural University, Zhengzhou 450046, China; College of Horticulture, Henan Agricultural University, Zhengzhou 450046, China; College of Horticulture, Henan Agricultural University, Zhengzhou 450046, China; College of Horticulture, Henan Agricultural University, Zhengzhou 450046, China; College of Horticulture, Henan Agricultural University, Zhengzhou 450046, China; College of Horticulture, Henan Agricultural University, Zhengzhou 450046, China; International Joint Laboratory of Henan Horticultural Crop Biology, College of Horticulture, Henan Agricultural University, Zhengzhou 450046, China; Research Center for Apple Engineering and Technology of Germplasm Innovation and Utilisation of Henan Province, College of Horticulture, Henan Agricultural University, Zhengzhou 450046, China; College of Horticulture, Henan Agricultural University, Zhengzhou 450046, China; International Joint Laboratory of Henan Horticultural Crop Biology, College of Horticulture, Henan Agricultural University, Zhengzhou 450046, China; Research Center for Apple Engineering and Technology of Germplasm Innovation and Utilisation of Henan Province, College of Horticulture, Henan Agricultural University, Zhengzhou 450046, China; College of Horticulture, Henan Agricultural University, Zhengzhou 450046, China; International Joint Laboratory of Henan Horticultural Crop Biology, College of Horticulture, Henan Agricultural University, Zhengzhou 450046, China; Research Center for Apple Engineering and Technology of Germplasm Innovation and Utilisation of Henan Province, College of Horticulture, Henan Agricultural University, Zhengzhou 450046, China; College of Horticulture, Henan Agricultural University, Zhengzhou 450046, China; International Joint Laboratory of Henan Horticultural Crop Biology, College of Horticulture, Henan Agricultural University, Zhengzhou 450046, China; Research Center for Apple Engineering and Technology of Germplasm Innovation and Utilisation of Henan Province, College of Horticulture, Henan Agricultural University, Zhengzhou 450046, China; College of Horticulture, Henan Agricultural University, Zhengzhou 450046, China; International Joint Laboratory of Henan Horticultural Crop Biology, College of Horticulture, Henan Agricultural University, Zhengzhou 450046, China; Research Center for Apple Engineering and Technology of Germplasm Innovation and Utilisation of Henan Province, College of Horticulture, Henan Agricultural University, Zhengzhou 450046, China; College of Horticulture, Henan Agricultural University, Zhengzhou 450046, China; International Joint Laboratory of Henan Horticultural Crop Biology, College of Horticulture, Henan Agricultural University, Zhengzhou 450046, China; Research Center for Apple Engineering and Technology of Germplasm Innovation and Utilisation of Henan Province, College of Horticulture, Henan Agricultural University, Zhengzhou 450046, China; College of Horticulture, Henan Agricultural University, Zhengzhou 450046, China; International Joint Laboratory of Henan Horticultural Crop Biology, College of Horticulture, Henan Agricultural University, Zhengzhou 450046, China; Research Center for Apple Engineering and Technology of Germplasm Innovation and Utilisation of Henan Province, College of Horticulture, Henan Agricultural University, Zhengzhou 450046, China; College of Horticulture, Henan Agricultural University, Zhengzhou 450046, China; International Joint Laboratory of Henan Horticultural Crop Biology, College of Horticulture, Henan Agricultural University, Zhengzhou 450046, China; Research Center for Apple Engineering and Technology of Germplasm Innovation and Utilisation of Henan Province, College of Horticulture, Henan Agricultural University, Zhengzhou 450046, China; College of Horticulture, Henan Agricultural University, Zhengzhou 450046, China; International Joint Laboratory of Henan Horticultural Crop Biology, College of Horticulture, Henan Agricultural University, Zhengzhou 450046, China; Research Center for Apple Engineering and Technology of Germplasm Innovation and Utilisation of Henan Province, College of Horticulture, Henan Agricultural University, Zhengzhou 450046, China; Henan Provincial Forestry Ecological Construction and Development Center, Henan Provincial Forestry Bureau, Zhengzhou 450003, China; College of Horticulture, Henan Agricultural University, Zhengzhou 450046, China; International Joint Laboratory of Henan Horticultural Crop Biology, College of Horticulture, Henan Agricultural University, Zhengzhou 450046, China; Research Center for Apple Engineering and Technology of Germplasm Innovation and Utilisation of Henan Province, College of Horticulture, Henan Agricultural University, Zhengzhou 450046, China; College of Horticulture, Henan Agricultural University, Zhengzhou 450046, China; International Joint Laboratory of Henan Horticultural Crop Biology, College of Horticulture, Henan Agricultural University, Zhengzhou 450046, China; Research Center for Apple Engineering and Technology of Germplasm Innovation and Utilisation of Henan Province, College of Horticulture, Henan Agricultural University, Zhengzhou 450046, China; College of Horticulture, Henan Agricultural University, Zhengzhou 450046, China; International Joint Laboratory of Henan Horticultural Crop Biology, College of Horticulture, Henan Agricultural University, Zhengzhou 450046, China; Research Center for Apple Engineering and Technology of Germplasm Innovation and Utilisation of Henan Province, College of Horticulture, Henan Agricultural University, Zhengzhou 450046, China

## Abstract

Apple replant disease (ARD) poses a major threat to global orchard productivity, yet its biological causes remain poorly understood. Although microbial dysbiosis in replant soils has been recognized as a major contributing factor, the specific pathogenic agents involved and the efficacy of synthetic microbial communities in mitigating ARD remain unclear. In this study, we integrated physiological, transcriptomic, metabolomic, and microbiome analyses to investigate the effects of replant soils on the growth of *Malus domestica* rootstock M26. Absolute quantification amplicon sequencing of 16S rRNA and ITS regions revealed a marked decline in rhizospheric microbial diversity in replant soils compared to fallow controls, accompanied by an enrichment of fungal genera such as *Fusarium*, *Aspergillus*, and *Acremonium*. Pathogenicity assays and seedling colonization experiments verified strong pathogenicity for five isolates—*Acremonium* sp., *Aspergillus niger*, *Fusarium solani*, *Macrophomina phaseolina*, and *Aspergillus stellatus*—implicating them as potential causal agents of ARD. High-throughput culturing and confrontation assays were used to isolate and screen candidate microbial antagonists. A synthetic microbiota (SynMs) composed of 12 bacterial strains and *Trichoderma* sp. was developed. Inoculation with SynMs significantly inproved plant height by 133% (*P* < 0.05) and total root length by 186% (*P* < 0.01), and effectively suppressed pathogen proliferation of the five pathogenic isolates in replant soils. Collectively, these findings identify key fungal pathogens underlying ARD and propose a sustainable microbiota-based strategy for its effective mitigation, offering both mechanistic insights and practical solutions for microbiome-informed orchard management.

## Introduction

Apple (*Malus* × *domestica* Borkh.) is one of the most widely cultivated fruit trees globally and is a major fruit crop in China. With increasing orchard age, tree senescence and susceptibility to diseases become more severe, necessitating the renewal of cultivars to sustain productivity and orchard health. However, the shortage of arable land often necessitates replanting on original sites, resulting in apple replant disease (ARD) [1, 2]. Apple seedlings affected by ARD exhibit root damage, stunted growth, sparse foliage, delayed development, reduced yield, and poor fruit quality [3, 4]. Globally, ARD poses a significant threat to the apple industry, leading to substantial economic losses [5].

**Figure 1:**
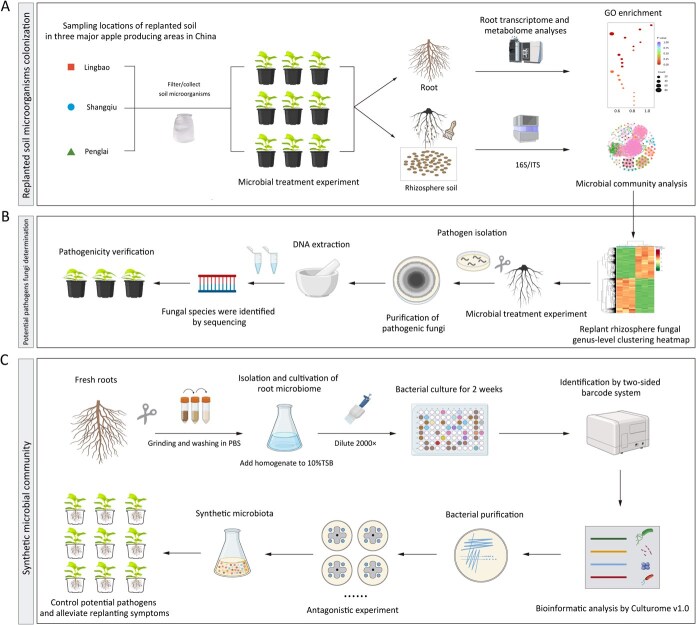
Microbial isolation and analysis of ARD (A); identification of potential pathogens (B); and construction of synthetic microbial community, the figure created withBioRender.com. accessed on 1 November 2023 (C).

Despite decades of investigation, the etiology of ARD remains elusive due to its complex, multifactorial nature. Abiotic factors such as allelopathic compounds and nutrient imbalances have been proposed [6, 7], but mounting evidence supports a dominant role of soil-borne microbial communities in ARD onset. Replanted soils showed a 40% reduction in beneficial bacteria, correlating with stunted root growth. So changes in the soil microbial community—especially in the composition and diversity of the rhizospheric microbiota—are considered the primary drivers of replant-associated stress [8, 9]. The microbial community of fungi, bacteria, and nematodes is significantly different in replanted soil [10]. The enrichment of pathogenic fungi in the apple rhizosphere, particularly *Fusarium solani*, is widely recognized as a major contributor to ARD [11, 12]. However, *Pythium* spp. are recognized as the primary causal agents of ARD in the UK, whereas *Pythium ultimum* and *Phytopythium litorale* are identified as the predominant pathogens in South Africa, demonstrating significant geographic variation of the pathogenic in ARD [13–15]. Therefore, specific causal microorganisms and their pathogenic mechanisms remain poorly defined, limiting the development of targeted and sustainable control strategies.

**Figure 2:**
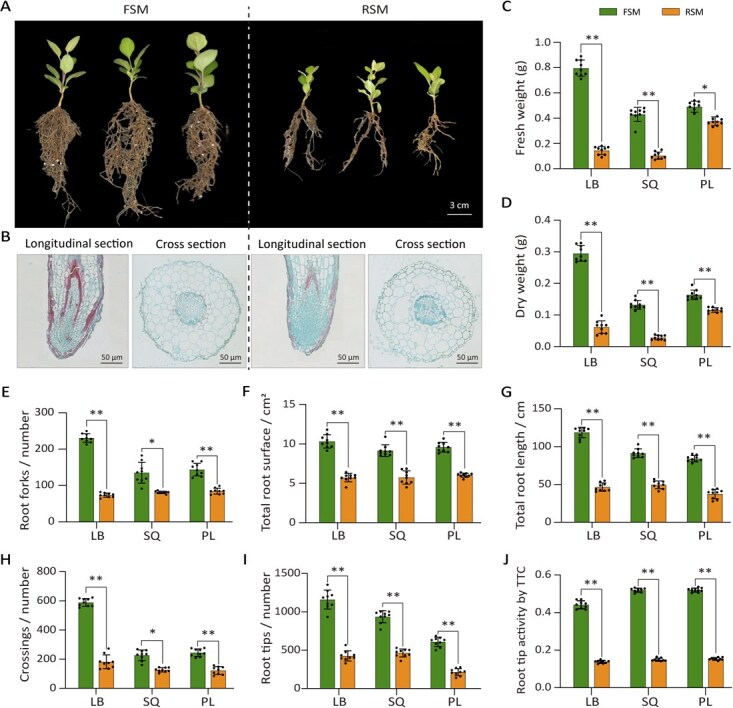
Microbiota transplantation reveals replant soil microbiomes inhibit apple seedling growth. (A) Phenotypes of saplings cultivated in FSM and RSM. (B) Microscopic observation of transverse and longitudinal sections of root tips grown in FSM and RSM after staining with paraffin section. The root fresh weight (C), root dry weight (D), number of root forks (E), total root surface area (F), total root length (G), number of root crossings (H), and root tips (I) of saplings grown in fallow and replant soil mixtures were determined. (J) TTC staining was used to measure root tip vitality. Significant differences were determined using the Mann–Whitney *U* test and indicated by asterisks (*n* = 12, ^*^P < 0.05; ^**^P < 0.01). The values represent the average of three biological replicates, and error bars indicate standard deviation (SD). FSM: microorganisms from fallow soil were added to sterilized soil, RSM: microorganisms from cultivated orchard soil were added to sterilized soil. LB: Lingbao orchard; SQ: Shangqiu orchard; PL: Penglai orchard.

Traditional strategies to ARD management, such as chemical fumigation and fungicide application, have shown some effectiveness but raise concerns regarding environmental toxicity and disruption of beneficial soil microbiota [16, 17]. Alternative methods, including biochar application and the use of biocontrol agents like *Bacillus* spp., offer more environmentally friendly solutions, but their efficacy is often inconsistent [18, 19]. Single-strain interventions may lack the ecological versatility and fail to restore a stable rhizospheric environment. Recent advances in synthetic microbiota ecology have demonstrated the efficacy of engineered communities in suppressing soil-borne phytopathogens, particularly fusarium wilt and root rot [20, 21]. The synthetic microbiota (SynMs) refers to a rationally designed consortium comprising two or more characterized microorganisms typically derived from native plant-associated environments [22]. The multispecies approach enhances both the structural resilience of the microbial community and the synergistic functionality of constituent strains. Compared to single-strain inoculants, SynMs offers greater potential for synergy and promote additional microbe–host interactions [23]. Soil-borne diseases are closely related to an imbalance in rhizospheric microbial composition and diversity [24]. SynMs constructed from root-associated microbes have been shown to inhibit the invasion of pathogens such as *Fusarium pseudograminearum* and *Magnaporthe oryzae*, thereby reducing disease severity [25–27]. Furthermore, SynMs consisting of diverse microbial members have demonstrated the ability to substitute for chemical inputs and improve salt–alkali stress tolerance in cotton [28, 29]. Thus, the development of complex SynMs represents a promising strategy for preventing or mitigating replant disease when establishing new plantings in previously cultivated orchard soils.

In this study, we aimed to uncover the microbial drivers of ARD and evaluate the efficacy of SynMs in ARD mitigation. The composition of the rhizospheric microbial community was analyzed through absolute quantification of microbial (16S rRNA and ITS) amplicon sequencing (Fig. 1). The results revealed a significant enrichment of fungal genera such as *Acremonium*, *Fusarium*, and *Aspergillus*, which were associated with inhibited roots development. To counter these adverse effects, high-throughput culturing and confrontation assays were employed to identify strains with broad-spectrum antifungal activity. We then assembled a functional SynMs from beneficial bacteria and fungi to suppress potentially pathogenic fungi in replant soil and further test its efficacy in mitigating ARD symptoms. Our findings provide novel insights into the microbial ecology underlying ARD and offer a sustainable microbiota-based strategy for disease management in perennial cropping systems.

## Results

### Microbiota derived from replant soil suppresses the growth of apple seedling

We first compared the soil nutrient profiles of apple orchard planting zones and adjacent fallow areas across three production regions. The soil organic matter, total nitrogen, available phosphorus, and available potassium were measured and no significant differences were observed between the fallow and replant soils (Fig. S1).

Next, soil microbial communities from replant soil or fallow soil were collected by elution and centrifugation, then resuspended in sterile water and reintroduced into presterilized growth substrates. Using this microbial transplantation approach, we prepared growth substrate with consistent physicochemical properties but distinct microbial communities, which were subsequently used to evaluate the effects of microbiota on apple root development. The tissue-cultured M26 apple plantlets were placed into the inoculated substrates with fallow soil microbiota (FSM) or replant soil microbiota (RSM) from three production areas (Lingbao, Shangqiu, and Penglai). After 30 days of cultivation, saplings grown in RSM exhibited significant dwarfed and shorter root systems compared to those grown in FSM (Fig. 2A). Microscopic examination of root cross-sections revealed abnormal cell division in the root elongation zone and root apical meristem (Fig. 2B). These cellular abnormalities were correlated with microbial shifts in RSM, suggesting a pathogen-mediated inhibition of root development.

Compared to saplings grown in the FSM, the biomass of plants cultured in RSM exhibited significant reductions (*P* < 0.01). Specifically, root length decreased by 52.53%, root dry weight by 75.42%, and root tip number by 53.47%, the consistent suppression patterns observed in the soils from the three different regions (Fig. 2C–I). Additionally, triphenyltetrazolium chloride (TTC) staining assay showed that root vitality in RSM-grown saplings was reduced by 70.2% (*P* < 0.01) relative to FSM-grown plants (Fig. 2J). These findings indicate that microbiota from replant soils significantly inhibit root elongation and lateral root formation in apple saplings.

### Differential transcripts and metabolites associated with cultivation in replant soil

To assess the biological impact of replant soil microbiota, we conducted the integrated transcriptomic and metabolomic analyses on root tissues of apple saplings grown in FSM and RSM from three production regions. A total of 581 differentially expressed genes (DEGs) were identified among plants grown in the different regional samples (Fig. S2A–C; Table S1). Gene Ontology (GO) enrichment analysis showed that these DEGs were primarily associated with reactive oxygen species (ROS) metabolism, responses to fungal and bacterial pathogens, defense response, regulation of primary metabolic processes, immune system processes, and root development (Fig. S2D). Notably, pathogenesis-related genes (e.g. *MD03G0016700* (Lectin Receptor-like Kinase 3, LECRK3), *MD09G0097300* (Receptor-like kinase containing the lysin motif), *MD03G0189300* (GRIM REAPER), and *MD02G0110900* (Mildew Resistance Locus O, MLO-like protein)) were upregulated in RSM (Table S1).

Metabolomic profiling identified 654 metabolites across all samples. Principal component analysis (PCA) showed distinct separation by soil sample, with the greatest variance observed between the FSM and RSM samples. PC1 and PC2 accounted for 61.62% and 12% of the variance, respectively (Fig. S2E). Cultivation in replant soils triggered distinct region-specific metabolic profiles relative to FSM controls (Fig. S2F and G; Table S2). Kyoto Encyclopedia of Genes and Genomes (KEGG) pathways analysis revealed significant alterations in arginine biosynthesis, amino acid biosynthesis, lipid metabolism, and sphingolipid metabolism in RSM-treated roots (Fig. S2H). Together, these findings indicate that replant soil microbiota induce coordinated changes in gene expression and metabolic activity in apple roots, which is closely related to differences in the soil microbial community.

### Microbial dysbiosis and enhanced fungal dominance in replant rhizospheres

**Figure 3:**
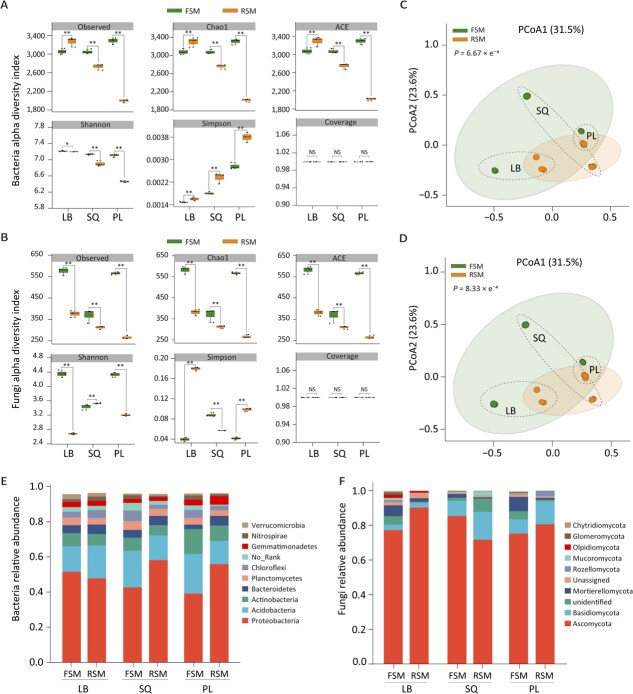
Analysis of the microbial communities in FSM and RSM rhizospheric soils. (A, B) Alpha diversity of bacteria and fungi species in rhizosphere soil inoculated with microorganisms from the different production regions (^*^P < 0.05; ^**^P < 0.01). (C, D) Principal coordinate analysis of the bacterial and fungal communities based on Bray–Curtis distance. (E, F) Relative abundance of dominant groups of bacteria and fungi in different production areas at the phylum level; species classification is based on RDP and UNITE databases. FSM: microorganisms from fallow soil were added to sterilized soil, RSM: microorganisms from cultivated orchard soil were added to sterilized soil. LB: Lingbao orchard; SQ: Shangqiu orchard; PL: Penglai orchard.

Next, we performed absolute quantitative amplicon sequencing targeting the 16S rRNA (bacteria) and ITS (fungi) regions to quantify rhizospheric microbial communities in fallow and replant soils. The results showed that both bacterial and fungal abundances in the rhizosphere of plants grown in the fallow soil mixtures were significantly higher than those grown in replant soils (*P* < 0.05). Moreover, soil origin from different regions influenced rhizospheric microbial diversity indices (Fig. 3A and B). Beta diversity analysis based on weighted UniFrac distance metrics showed clear clustering patterns of rhizospheric bacterial and fungal communities between fallow and replant soils (Fig. 3C and D). Notably, soil treatment (fallow vs replant) accounted for 31.5% of the total variation in community composition, while geographical origin contributed an additional 23.6%. These findings support the microbial assembly theory that deterministic processes predominantly shape microbial communities under replant conditions. Specifically, taxonomic profiling indicated significantly lower relative abundances of species within the phyla *Actinobacteriota* and *Firmicutes* in the rhizosphere of apple grown in replant soil compared to those in fallow soils (Fig. 3E, Fig. S3A). In contrast, fungal communities in replant soil exhibited significantly higher relative abundance of *Ascomycota* and *Basidiomycota* (Fig. 3F, Fig. S3B), with pronounced increases in *Fusarium* and *Aspergillus* genera (Fig. S4).

**Figure 4:**
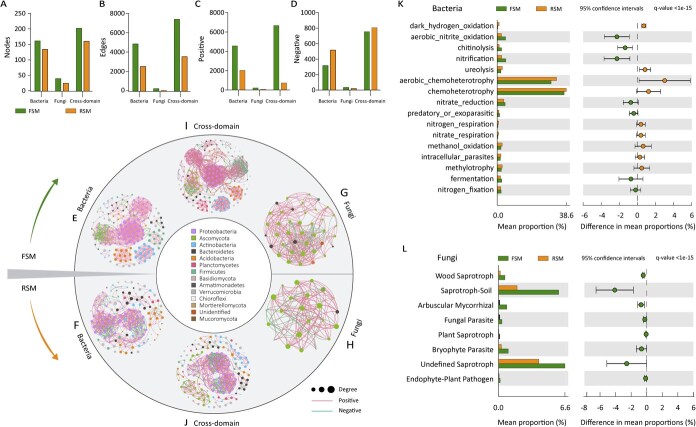
Ecological network map and functional differences of the rhizospheric microbial communities of plants grown in FSM and RSM. Number of (A) nodes, (B) edges, (C) positive correlation connections, and (D) negative correlation connections in the different microbial networks. Rhizospheric bacterial symbiotic network maps for bacteria (E, F) and fungi (G, H) from saplings grown in fallow and replanted soil mixtures. (I, J) Map of interdomain symbiotic networks of bacteria and fungi in the rhizospheres of fallow and replanted saplings. Node size represents node connectivity, node color represents phylum level, different nodes represent different genera. (K, L) Extended error bar plot identifying differences between mean proportions in FSM and RSM are shown for FAPROTAX and funguild functional groups. The plot was generated in STAMP; the difference between the groups showed a 95% confidence interval, and only the groups with *q*-values <0.05 (Welch’s *t*-test) after FDR correction were shown. FSM: microorganisms from fallow soil were added to sterilized soil, RSM: microorganisms from cultivated orchard soil were added to sterilized soil. LB: Lingbao orchard; SQ: Shangqiu orchard; PL: Penglai orchard.

Based on the core microbiota identified across the three production regions, we constructed the bacterial, fungal, and cross-domain symbiotic networks to evaluate the interaction patterns within the rhizospheric microbial communities of the fallow and replant soil (Fig. S5; Table S3). The results showed that the microbial community in the fallow rhizosphere was more diverse and structurally complex, exhibiting a greater number of nodes and edges as well as more synergistic interactions compared to the replant rhizospheres (Fig. 4A and B). In the replant rhizospheres, the proportion of positively correlated connections between bacteria and fungi taxa was markedly reduced, indicating a shift toward antagonistic interactions (Fig. 4C and D). Overall, microbial networks in replant soils exhibited diminished complexity, with increased negative correlations involving key taxa such as *Bacillus* and *Rhizobium* (Fig. 4E and F). In contrast, the relative abundance of *Aspergillus* and *Fusarium* showed strong positive correlations in the replant rhizospheres, while *Trichoderma* displayed a negative correlation (Fig. 4G and H). These results suggest that the enrichment of potentially pathogenic fungi such as *Fusarium* and *Aspergillus* may underlie the replant disease symptoms observed in apple saplings. Additionally, cross-domain network exhibited a greater number of connections than intradomain bacterial or fungal networks in both fallow and replant soils, indicating that interkingdom interactions play a dominant role in shaping rhizospheric microbial community structure (Fig. 4I and J).

Functional profiling of the bacterial community revealed that rhizospheres associated with fallow soil were enriched in functions such as aerobic nitrite oxidation, nitrification, chitinolysis, and fermentation. In contrast, rhizospheres in replant soil showed higher functional potentials related to dark hydrogen oxidation, aerobic chemoheterotrophy, ureolysis, chemoheterotrophy, and methanol oxidation (*P* < 0.05; Fig. 4K). Similarly, the fungal community in fallow-soil rhizosphere exhibited a greater abundance of Endophyte-Litter Saprotroph-Soil Saprotroph, Arbuscular Mycorrhizal, Undefined Saprotroph, and Ectomycorrhizal-Fungal Parasite-Plant Pathogen-Wood Saprotroph-type species (*P* < 0.05; Fig. 4L). These results indicate that replanting alters the functional landscape of rhizosphere microbiota, likely reducing beneficial processes related to nutrient uptake and mutualistic symbioses. Such changes may compromise nutrient uptake efficiency and weaken plant defenses against soil-borne pathogens.

### Fungal microorganisms enriched in replant rhizospheres include potential pathogens

**Figure 5:**
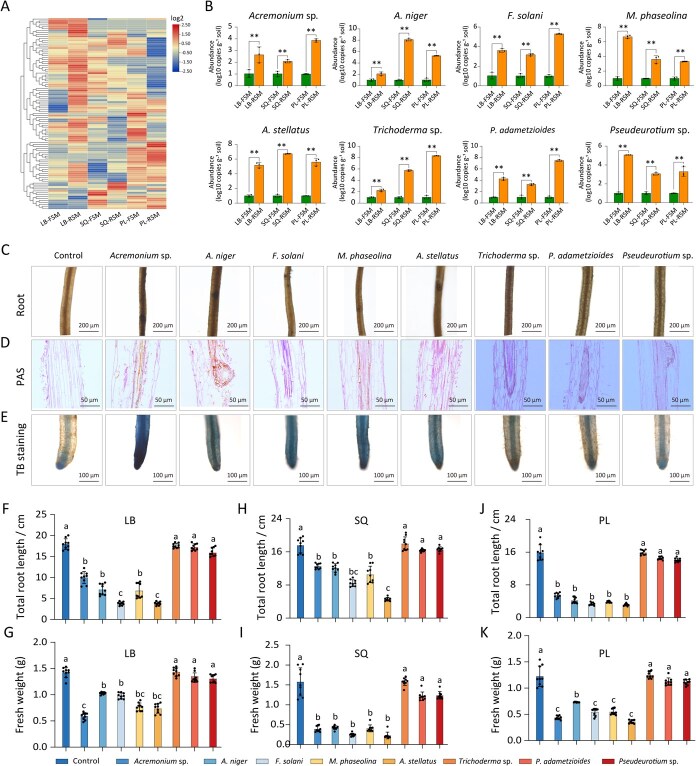
Identification of the main pathogens linked to replantation disease. (A) Genus-level clustering heatmap of fungus from the rhizospheres of apple plants grown in FSM and RSM (LB, SQ, and PL). (B) Quantitative reverse transcription polymerase chain reaction (qPCR) detection of differential fungi (^*^P < 0.05; ^**^P < 0.01). (C) Microscopic observation of roots after infection by pathogens. (D) Observation of root sections stained with PAS 30 days after microbial inoculation. (E) Observation of root tip vitality after microbial inoculation using viability staining. (F–K) Total root length and root fresh weight of plants inoculated with a single microbial isolate and grown in sterilized soil for 4 weeks. Average value of three biological replicates, the data are presented as mean ± SD (*n* = 9, *P* < 0.05).

To investigate fungal taxa potentially associated with ARD, fungal species were analyzed using high-throughput sequencing and clustered via heatmap. Notably, species such as *Fusarium* and *Acremonium* were significantly enriched in the rhizosphere of replant soils (Fig. 5A). Quantitative polymerase chain reaction (qPCR) targeting key fungal taxa with pronounced abundance differences confirmed the sequencing-based results across all three production regions (Fig. 5B).

While sequencing and qPCR analyses of the fungal community revealed compositional shifts, pathogenicity required experimental validation. To this end, eight fungal species were isolated from rhizospheric soils in different production regions (Table S4). To assess their pathogenic potential, roots of young *Malus domestica* M26 rootstocks were immersed in spore suspensions of each fungal isolate. Thirty days post-inoculation, plants inoculated with *Acremonium* sp., *Aspergillus niger*, *F. solani*, *Macrophomina phaseolina*, and *Aspergillus stellatus* developed brown spots on the root surface compared to the uninoculated controls. Microscopy revealed that conidia and hyphae progressively penetrated into the vascular cylinder, forming brown necrotic zones (Fig. 5C, Fig. S6A and D). Periodic acid–Schiff (PAS) staining of root sections showed ruptured, detached, and deformed epidermal and cortical cells, as well as irregular cell arrangement. Extensive fungal hyphae were observed invading cortical cells and vascular bundles (Fig. 5D, Fig. S6B and E). Toluidine blue (TB) staining further revealed a marked reduction in root tip vitality (Fig. 5E, Fig. S6C and F). These results confirm that several fungal taxa enriched in replant rhizospheres exhibit pathogenic traits likely contributing to the development of ARD symptoms.

Thirty days after fungal inoculation, *Penicillium oxalicum* and *Pseudeurotium* sp. exhibited weak or pathogenic effects, as no root lesions were observed and sapling biomass showed no significant differences compared to the control group. In contrast, inoculation with *Trichoderma* sp. slightly promoted sapling growth. PAS staining revealed relatively intact epidermal and cortical cells, with no visible hyphal invasion (Fig. 5D, 7th panel). Conversely, inoculation with *Acremonium* sp., *A. niger*, *F. solani*, *M. phaseolina*, and *A. stellatus* significantly inhibited the growth of M26 apple rootstock. Total root length was reduced by 69.5% (*P* < 0.05), and fresh weight decreased by 84.4% (*P* < 0.01) compared to the control (Fig. 5F–K). These results suggested that these fungi are strongly associated with replant symptoms under controlled experimental conditions.

### Constructing synthetic microbial communities to counter apple replant disease

**Figure 6:**
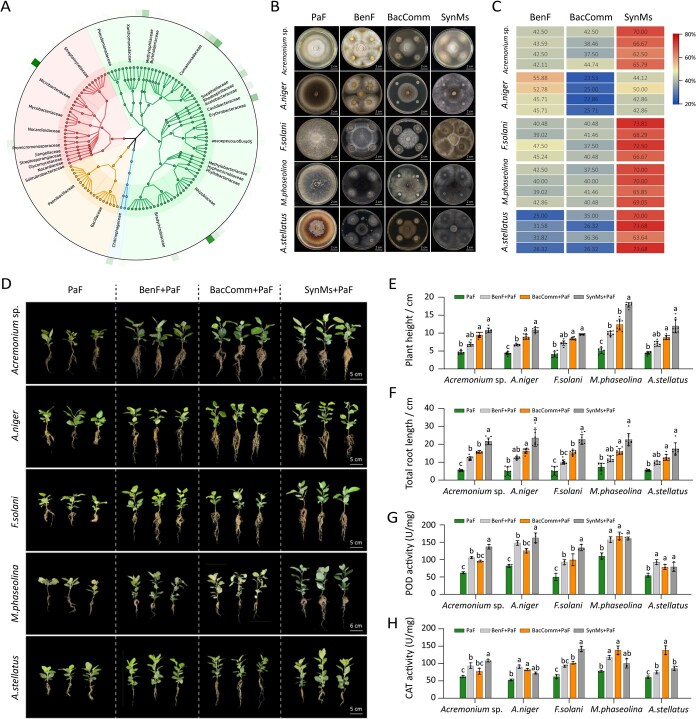
Synthetic microbial community construction and verification of therapeutic effects. (A) Taxonomic composition of cultivable bacteria. (B) Observation of the antagonistic effects of different microbial communities against five fungal pathogen isolates on PDA culture plates. (C) Inhibition rates of different microbial communities against pathogens on PDA culture plates. (D) Representative images of apple saplings inoculated with BenF, bacterial communities (BacComm), and synthetic microbial communities (SynMs), and subsequently inoculated with different representative pathogens. Physiological indicators of saplings grown with different microbial communities and challenged with fungal pathogens reflect the therapeutic effects, including plant height (E), total root length (F), POD enzyme activity (G), and CAT enzyme activity (H). *P* < 0.05, Kruskal–Wallis test with Dunn’s *post hoc* test, *n* = 9 biologically independent plants. PaF: Pathogenic Fungi; BenF+PaF: Beneficial Fungi + Pathogenic Fungi; BacComm+PaF: Bacterial Community + Pathogenic Fungi; SynMs+PaF: Synthetic Microbial community + Pathogenic Fungi.

Recent studies have demonstrated that synthetic microbial communities (SynMs) can not only suppress plant pathogens but also enhance plant growth and stress tolerance [25–27]. To develop an effective SynMs strategy for the control of ARD, we employed high-throughput cultivation and double-labeling strategies to isolate 1409 bacterial strains from the rhizosphere of healthy apple plants. These isolates were taxonomically classified into 129 distinct bacterial species (Fig. 6A, Fig. S7). For each species, one to two representative strains were randomly selected for antagonistic screening. The antagonistic potential of these bacterial isolates was evaluated using dual-culture assays on potato dextrose agar (PDA) plates. Twelve bacterial species exhibited significant inhibitory activity against ARD-associated pathogens, with inhibition rates ranging from 25.71% to 70.73% (Fig. S8). These strains were subsequently selected as core candidates for SynMs construction.

**Figure 7:**
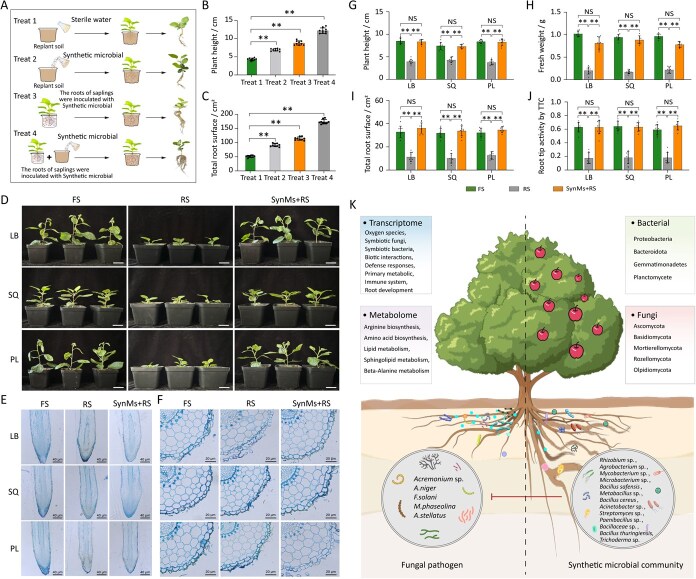
The effects of synthetic microbial communities on apple plants cultivated in replant soils from various production areas. (A) Comparative analysis of different inoculation methods of synthetic microbiota in preventing ARD. (B, C) Following the different inoculation methods, comparisons were made on plant height and total root surface area. (D) Representative images of apple saplings planted in fallow soil, replant soil, and replant soil after inoculation with synthetic microbial communities. (E, F) Microscopic observation of longitudinal section and transverse section of semithin sections on the control effect of synthetic microbiota on ARD. (G–J) Comparison of physiological indicators reflect the effects of synthetic microbial communities in replant soils from different production areas, including plant height (G), fresh weight (H), total root surface (I), and root tip vitality as determined by TTC (J). *P* < 0.05, Kruskal–Wallis test with Dunn’s *post hoc* test (*n* = 9), biologically independent plants. (K) Summarization of the differential changes in rhizospheric microbial communities and their impact on apple saplings growing in replant soils, the figure created with BioRender.com. accessed on 1 November 2023. The schematic representation illustrates the beneficial effects of the synthetic microbial community. FS: planting saplings in fallow soil; RS: replanting saplings in the orchard soil; SynMs+RS: inoculating the roots of saplings with the synthetic microbial community and then planting them in the replant soils from different growing areas. LB: Lingbao orchard; SQ: Shangqiu orchard; PL: Penglai orchard.

Given that *Trichoderma* sp. are widely recognized for their antifungal properties [30], we further assessed their efficacy in suppressing ARD pathogens using *in vitro* dual-culture assays. The results showed that *Trichoderma* sp. strains inhibited pathogens growth by 25%–55.88% (Fig. 6B and C), supporting their inclusion in SynMs for potential synergistic effects. Therefore, we propose constructing a SynMs comprising *Trichoderma* sp. and bacteria to achieve synergistic biocontrol effects. Compatibility assays revealed that 12 bacterial strains were mutual compatibility (Fig. S9). Moreover, *Trichoderma* sp. exhibited stable coexistence with eight of the bacterial isolates, while delayed compatibility was observed with four strains (including *Rhizobium* sp.) over extended co-culture periods (Fig. S10). Based on these findings, we constructed distinct SynMs and evaluated their efficacy. A SynMs consisting of the 12 bacterial strains and *Trichoderma* sp. exhibited robust inhibition against ARD pathogens, with inhibition rates ranging from 42.86% to 73.81%, significantly outperforming bacteria-only SynMs (Fig. 6B and C). These results lay a solid foundation for subsequent *in planta* validation of SynMs-mediated ARD suppression.

Construction of SynMs is a critical first step toward functional validation, with field applications requiring further testing. Based on the results of dual-culture assays on PDA plates, we evaluated the biocontrol efficacy of three treatments against phytopathogen-infected plants: (i) *Trichoderma* sp. alone (BenF), (ii) a consortium of 12 bacterial isolates (BacComm), and (iii) a SynMs comprising of the 12 bacterial strains and *Trichoderma* sp. (SynMs). The SynMs treatment showed significantly stronger pathogen suppression than other treatments, which aligns with the results of the *in vitro* plate assays (Fig. 6D). Compared to the control, preventive application of SynMs significantly promoted seedling development, increasing root length and shoot biomass by 169.8% and 171.33%, respectively (*P* < 0.05), alongside elevated peroxidase (POD) and catalase (CAT) activities (Fig. 6E–H, Fig. S11).

Further comparison of different inoculation strategies revealed that combined root and soil inoculation of SynMs was significantly more effective than either method alone (Fig. 7A–C). This synergistic inoculation strategy consistently enhanced seedling performance across all three production regions tested (Fig. 7D). Microscopic analysis confirmed that SynMs treatment stimulated root meristematic activity and promoted cellular division and differentiation in replant soil conditions (Fig. 7E and F). Compared to untreated controls in replant soil, the biomass of the young apple plants treated with SynMs significantly increased (*P* < 0.05), with plant height rising by 133.3% and total root length by 185.5% (*P* < 0.05; Fig. 7G–J, Fig. S12). Moreover, the abundance of pathogenic fungi in the rhizosphere was markedly reduced following SynMs application (Fig. S13). Together, these results demonstrate that rationally designed SynMs can suppress fungal pathogens, alleviate replant disease symptoms, and significantly promote the growth and vigor of apple seedlings under controlled experimental conditions (Fig. 7K).

## Discussion

### Pathogenic soil microbiota in established orchards inhibit root development

Physiological and biochemical parameters are important indicators of internal and external factors influencing plant growth. In M26 apple rootstocks grown in replant soils from established orchards, key physiological indexes, such as number of root tips, total root length, and root tip activity, were significantly reduced compared to those grown in fallow soils. These observations suggest that young apple plants experience stress under replant soil conditions. Analysis of soil physicochemical properties revealed no significant differences among the soils from different production regions, indicating that variations in microbial communities, rather than abiotic factors, may underlie the observed differences in seedling development. This inference is consistent with previous findings linking replant disease to rhizospheric microbiota rather than soil nutrients [9].

Transcriptomic profiling of apple root revealed significant enrichment in pathways related to bacterial and fungal responses as well as ROS metabolism, indicating the activation of defense-related signaling under microbial stress. Lipids, as key components of cellular membranes, are central to plant–microbe interactions and perception–defense processes. Metabolomic analysis showed marked differences in lipid-related metabolites between plants grown in replant and fallow soils. Previous studies have shown that lipid-mediated signaling can regulate both plant growth and resistance to biotic stress through interactions with the soil microbiome [31]. Taken together, under conditions of uniform orchard management and comparable soil physicochemical characteristics, our results suggest that pathogenic soil microbiota in replant soils may impair nutrient uptake and disrupt root physiology, thereby triggering transcriptomic and metabolic responses in apple seedlings.

### Soil microorganisms in replant soil alter the rhizospheric microbial profile of apple saplings

Recent studies have demonstrated that replant soils reduce rhizospheric microbial diversity, disrupt microbial interactions, and impair nutrient cycling and uptake, thereby contributing to replant disorders [6, 32]. Consistent with these findings, absolute quantitative amplicon sequencing in our study revealed a significant decrease in both bacterial and fungal diversity in the rhizosphere of apple saplings grown in replant soils. Similar observations have been reported in other replant-prone systems such as tomato, peach, and *Panax notoginseng*, where repeated planting altered rhizosphere microbiota composition and reduced community diversity [33–35]. These findings support the hypothesis that continuous monoculture imposes biological stress on seedlings, which inhibits their growth—a pattern also reported in previous studies [5, 36, 37].

Disruptions in nutrient cycling were further exacerbated by shifts in microbial community composition. Notably, the phylum *Acidobacteria*, which consists of oligotrophic bacteria that are often negatively correlated with soil fertility [38], was markedly enriched in replant soils. Specifically, Gp6, Gp16, and Gp17 exhibited 2.3-fold higher relative abundance in replant rhizospheres (Fig. S4), potentially creating an ecological niche conducive to pathogen establishment. This microbial imbalance motivated subsequent pathogen screening to identify the primary biotic stressors.

Several pathogenic fungal genera, including *Fusarium*, *Aspergillus*, *Acremonium*, *Pseudeurotium*, and *Tausonia* exhibited increased abundance in continuously cropped soils [4, 39]. The enrichment of these taxa likely triggers defensive responses from biocontrol-associated bacteria. Co-occurrence network analysis revealed weakened microbial network connectivity in replant rhizospheres, indicative of reduced community stability that may facilitate pathogen invasion. Notably, well-known biocontrol taxa such as *Paenibacillus* and *Bacillus* exhibited intensified negative correlations with pathogenic fungi, consistent with their antagonistic ecological roles [19, 40–42]. Similarly, the biocontrol fungus *Trichoderma* showed negative associations with *Mortierella*, *Acremonium*, and other fungal taxa within replant soils. Moreover, the overall proportion of saprotrophic fungi, which are key contributors to rhizosphere multifunctionality, was reduced, suggesting a decline in beneficial fungal functions [43]. Taken together, these observed microbial shifts, particularly the proliferation of oligotrophic *Acidobacteria* and pathogenic *Fusarium*, prompted us to systematically identify the specific fungal pathogens responsible for ARD symptoms.

### Identification of fungal genera causing replanting disease symptoms in apple saplings

Our study represents a significant advancement in the identification of specific fungal pathogens responsible for ARD, including several genera with previously limited experimental validation in the context of ARD. Based on the identification of changes in the microbial communities of replant soil, we employed a three-pronged approach (sequencing analysis, microbial isolation, and pathogenicity assays) to pinpoint the key pathogenic agents driving replant disease. This targeted strategy revealed that *Acremonium* sp., *A. niger*, *F. solani*, *M. phaseolina*, and *A. stellatus* exhibit high pathogenicity toward apple rootstock M26. While previous studies have recognized *Fusarium* spp. as major ARD pathogens [12], our findings further confirm the strong pathogenicity of *F. solani* and suggest potential synergistic interactions with other fungi*.* Importantly, our results expand the spectrum of known ARD-associated fungi by demonstrating the pathogenic role of *A. niger*, *Acremonium* sp., and *Phoma* spp., which have been rarely reported or experimentally verified in this context. Notably, *Aspergillus flavus* has been negatively correlated with young apple plant growth, and *Phoma* spp. are known to cause black rot in crops such as watermelon and stevia [44, 45]. Microscopic analysis of infected roots revealed hallmark symptoms, including brown lesions, ruptured epidermal cells, and inhibited root elongation, further supporting the pathogenic roles of these fungi. In addition, since root exudates are known to regulate rhizosphere microbial communities and can recruit beneficial organisms pathogen suppression [46, 47], we hypothesize that replanting in established orchards may alter root exudation patterns in young apple plants. This possibility warrants further investigation. Collectively, fungal inoculation and plant biomass analyses confirm a causal relationship between specific pathogenic fungi and replant symptoms. These findings provide a critical foundation for the development of effective ARD management strategies targeting fungal pathogens.

### Synthetic microbiota diminishes apple replanting disease

Having established the pathogenic mechanisms and identified key fungal culprits, given the complex microbial interactions revealed by co-occurrence network analysis (Figs 4 and 5), we then sought to develop a biocontrol strategy using synthetic microbial consortia that could counteract both the nutrient-depleting bacteria and fungal pathogens identified in previous analyses. Although synthetic microbiota has been applied in managing wilt and root rot disease in other crops, the contribution of a bacterial–fungal synthetic microbiota to ARD management remains poorly understood. Apple replanting disease is a globally recognized problem. Compared to single-strain inoculants, a multimember synthetic microbiota enhances rhizosphere colonization, increases microbial diversity, and reduces the risk of functional loss within the microbial community [48]. Accordingly, we isolated rhizosphere microorganisms from healthy apple roots, and screened 12 bacterial strains that, when combined with *Trichoderma* sp., not only suppressed pathogenic fungi but also improved the apple seedling growth in replanted soil. These findings improve our understanding of the interactions between bacterial and fungal communities in plant health and provide a foundation for an effective and sustainable biocontrol strategy.

Inoculation with the synthetic microbiota highlighted the importance of functional interactions between microbial taxa within the rhizosphere. Some bacterial strains, such as *Bacillus cereus* and *Bacillus paeniformis*, are syntrophic organisms capable of degrading organic matter through enzymatic activity [49], while members of the phylum *Proteobacteria*, including *Rhizobium* and *Agrobacterium*, may promote nutrient uptake by fixing nitrogen [40]. The fungal genus *Trichoderma* is also known to enhance plant growth and induce systemic resistance [30, 50]. Notably, *Rhizobium* and *Bacillus* strains in SynMs specifically counteracted the enriched *Acidobacteria* observed in replant soils, while *Trichoderma* sp. directly antagonized the pathogenic. These findings are consistent with previous reports showing that fungal and bacterial members of microbial consortia suppress pathogens via distinct but complementary mechanisms [51], offering a robust and sustainable solution to replant disease. While SynMs demonstrated significant efficacy under controlled conditions, further validation in field environments is essential to assess their practical performance across diverse orchard systems and soil types.

## Conclusion

Replanting apple saplings in aged orchard soil significantly suppressed plant growth and reduced rhizospheric microbial diversity, which facilitated the enrichment of pathogenic fungi (including *Acremonium* sp*.*, *A. niger*, *F. solani*, *M. phaseolina*, and *A. stellatus*). By constructing a synthetic microbiota through the targeted isolation of antagonistic bacterial and fungal strains, we effectively restored plant resistance against these pathogens. The introduced microbial consortium not only restructured the rhizospheric microbial community but also markedly improved sapling growth and viability in replanted soils. These findings provide a promising and sustainable alternative to chemical fumigation for the management of ARD.

## Materials and methods

### Soil samples

Soil samples were collected from aging apple orchards (cultivated for >10 years) located in three different regions: Lingbao County, Sanmenxia City, Henan Province (LB; 30°31′N, 110°53′E); Zhaolou Village, Dahou Township, Shangqiu City, Henan Province (SQ; 34°04′N, 115°50′E); and Guanzhuangzi Village, Penglai City, Shandong Province (PL; 37°46′N, 120°53′E). Each orchard had been established for >20 years, and replant disease symptoms were observed in newly planted apple saplings 1 year after replanting. Soil collected from these sites was designated as replant soil (RS). For each orchard, nine composite samples were collected from the rhizosphere (10–40 cm depth, within a 50 cm radius of symptomatic tree trunks) for further analyses and assays. In parallel, soil samples collected from uncultivated inter-row areas within the same orchards were designated as fallow soil (FS). The soil was analyzed using Qiu’s method [52] to determine the organic matter, total nitrogen, available phosphorus, and available potassium.

### Soil treatments, soil and plant analyses

The tissue-cultured M26 apple plantlets were first cultivated on one-half MS medium for 30 days to induce rooting [53]. Rooted plants were subsequently transferred to a sterilized substrate (substrate:vermiculite:perlite was mixed at 3:2:1, sterilized at 120°C for 15 min) for further growth.

Microbial communities from replant and fallow soils were extracted using the following protocol. First, soil impurities were removed by passing soil through a 200-mesh sieve. Each 500 g of sieved soil was then suspended in 1 l of sterile water, thoroughly mixed, and the supernatant collected. This extraction procedure was repeated three times to maximize microbial yield. The combined supernatants were centrifuged at 4000 × *g* for 10 min, and the resulting microbial pellet was resuspended in 200 ml of sterile water to generate a concentrated microbial suspension. This suspension was then thoroughly mixed with presterilized cultivation soil and used to grow *M. domestica* M26 apple seedlings. Soil samples from three production regions (Lingbao, Shangqiu, and Penglai) were processed in parallel, and microbial suspensions from each were combined with standardized substrate to ensure consistent physicochemical properties across all treatments.

After 30 days of pots cultivation, root morphological traits, including root length, root tip number, and surface area, were measured using a root scanner (GXY-A, China). Statistical analysis was performed using the Mann–Whitney *U* test in GraphPad Prism. For histological analysis, root tips were paraffin-embedded, sectioned, and stained with safranin-fast green. Root activity was assessed using TTC reduction method, as described previously [54]. To prepare samples for microbial analysis, large soil particles adhering to the root surface were were first removed. Rhizosphere soil was then detached from the root zone by rinsing with 20 ml of sterilized distilled water, followed by centrifugation. The resulting microbial pellet was flash frozen with liquid nitrogen and stored at −80°C for subsequent DNA extraction and microbial sequencing analysis. Root samples were washed again with distilled water, blotted dry with sterilized paper, frozen in liquid nitrogen, and then stored at −80°C for root transcriptome sequencing and primary metabolism analyses. Each treatment had three biological replicates, with each replicate consisting of roots pooled from three independent apple seedlings.

### Root transcriptome and metabolome analyses

Total RNA from root samples was extracted using the TRIzol method, and RNA integrity and purity were assessed by agarose gel electrophoresis and NanoPhotometer spectrophotometry (Thermo Fisher Scientific, USA). The total RNA (≥1 ug) was sequenced using the Illumina NovaSeq 6000 (MetWare, Wuhan, China). The raw data was filtered using the fastp tool to remove sequences with adapters, and the clean reads were aligned to the reference genome. Gene annotation was performed using KEGG [55] and GO databases [56]. Gene expression levels were quantified using the FPKM algorithm [57]. Differential gene analysis was conducted between different groups, with a significance threshold set at *P* ≤ 0.05 and |log2 fold change| > 1.

In parallel, primary metabolomics analysis of the root system was performed using a ultra-performance liquid chromatography-tandem mass spectrometry (UPLC-MS/MS, ExionLC™ AD/QTRAP 6500) detection platform (MetWare, Wuhan, China) and a self-constructed metabolite database of MetWare (MWDB). Qualitative and quantitative analysis of metabolites was conducted using standard multiple reaction monitoring (MRM) chromatography and MultiQuant software. Differential metabolites with *P-*values ≥1 and |log2 fold change| ≥ 1 were selected, and KEGG pathway enrichment analysis was subsequently performed.

### Absolute quantitative sequencing of rhizospheric microorganisms and data processing

DNA was extracted from each rhizospheric soil sample using the FastDNA® SPIN Kit for Soil DNA Extraction (MP Biomedicals, Santa Ana, CA, USA) following the manufacturer’s instructions. DNA quality was evaluated by 1.5% agarose gel electrophoresis and quantified using a Qubit 3.0 fluorometer (Thermo Fisher Scientific, USA), then diluted to a final concentration of 10 ng/μl in sterile water. Three biological replicates were prepared per treatment. The 16S rRNA and ITS gene libraries were constructed and subjected to absolute quantitative sequencing analysis using the Illumina 2 × 250 bp paired-end sequencing platform (Genesky Biotechnologies Inc., China) [58]. Raw data quality was evaluated using the QIIME 2 software [59], and reads were denoised and filtered using DADA2 (v. 1.8) to generate feature tables and representative Amplicon Sequence Variants (ASVs) [60]. A standard curve was constructed using reference DNA with known copy numbers to calculate the absolute abundance of each ASV in the sample. The UPARSE v7.0.1001 software was used to assign sequences with a similarity ≥97% to the same operational taxonomic units (OTUs) [61], and taxonomic classification of ASVs was performed in QIIME 2 using the RDP (for 16S) and UNITE (for ITS) databases.

Alpha diversity indices, including observed species, Chao1, ACE, Shannon, Simpson, and Coverage, were calculated based on ASV tables [62]. Beta diversity was calculated using Unifrac distance, and Principal Coordinate Analysis (PCoA) was performed using FastUnifrac [63]. To examine differences in rhizospheric microbial communities among replant soil samples, Venn diagrams were constructed to identify shared and unique taxa among the six treatment groups. Co-occurrence network analysis was then conducted using the ggcluster R package [64], and topological properties including edge count, average path length, average degree, and vertex count were calculated (*r* > 0.70, *P* < 0.05). Each node represented an ASV, and each edge represented the correlation between two nodes. The network relationships were visualized using the Gephi platform [65].

Functional prediction of bacterial communities was performed using the FAPROTAX database [66], while fungal functional guilds were annotated using the FUNGuild database [67]. Finally, the STAMP software was employed for assessing significant differences in functional predictions among different regions [68].

### Isolation of potential pathogenic fungi related to replantation disease

To elucidate the etiology of replant disease, fungi were isolated from the roots of apple plants grown in replant soils, with a focus on taxa identified as enriched in the rhizospheric microbiota. Purification of different fungi by morphology was performed on PDA plates [69]. Purified single-strain mycelium was collected, and DNA extraction was carried out using a fungal DNA extraction kit (Sangon, shanghai, China). PCR amplification and sequencing were conducted using universal primers ITS1 and ITS4 (Table S5). The obtained sequences were compared to the NCBI database for identification of the fungal species. Eight fungal species with significantly differential abundance in the replant rhizosphere were selected for pathogenicity verification.

The eight fungal isolates were cultured from a single spore (Table S4). Spore suspensions were prepared from 7-day-old cultures in potato dextrose broth (PDB), quantified using a hemocytometer, and diluted to a concentration of 10^6^ conidia per gram of sterile soil. Apple roots were inoculated with the fungal suspensions using the root immersion method, while plants in the control group were treated with sterile water. Seedlings were transplanted into sterilized substrate (peat:vermiculite:perlite, mixed at a 3:2:1 ratio and autoclaved at 120°C for 15 min) and maintained in a growth chamber for 4 weeks. Disease symptoms were recorded, and root physiological parameters were measured as described previously. Fungal pathogenicity was evaluated based on the presence of root browning with characteristic necrotic lesions. PAS staining was performed on the root tissue sections to observe the fungal infection of the root tips. Using Koch’s postulates, fungi were re-isolated from symptomatic roots and identified at the genus level for each isolate.

### High-throughput isolation, culture, and identification of bacteria

Fresh apple root samples (3 g) were collected from healthy plants for bacterial isolation. According to published high-throughput isolation and cultivation methods, a double-labeled two-step PCR amplification method was employed to identify the 16S rRNA gene of each bacterial isolate [70]. The PCR products from each plate were pooled and purified using the NucleoSpin beads purification kit. The purified and concentrated mixture was subjected to Illumina sequencing using 1500 ng of the amplified product. The sequencing data were analyzed using an open-access data analysis pipeline (https://github.com/YongxinLiu/Culturome) to generate ASVs and assign taxonomic annotations to each isolate (Table S6).

### Screening for antagonistic strains

Bacterial strains were purified using one-half Trypticase Soy Broth (TSB) solid medium for subsequent antagonistic tests against pathogenic fungi. For confrontation assays, each pathogenic fungus was inoculated in the center of PDA plates, while the bacterial strains were inoculated 3 cm out from the center. PDA plates without bacterial inoculation were used as control. After 7 days of incubation, the inhibitory effect of the bacteria was evaluated, and the inhibition rate was calculated to select antagonistic strains. Similarly, antagonistic tests were conducted for the isolated *Trichoderma* fungi, and the inhibition rates against pathogenic fungi were calculated. To assess their co-culture compatibility, on one-half TSB solid agar plates, two distinct bacterial strains were streaked in perpendicular cross-streaks to form a ‘+’ intersection. Simultaneously, dual-culture assays were conducted between *Trichoderma* sp. and each of the 12 bacterial isolates. Then the inhibition rates of the SynMs consisting of the 12 bacterial strains and *Trichoderma* sp. against pathogenic fungi were calculated on PDA plates.

### Constructing synthetic microbial communities to reduce replant symptoms in apple root

All bacteria were cultured in TSB for 4 days. After centrifugation at 4000 × *g* for 10 min, the bacterial pellets were resuspended to a final OD_600_ of 0.02, corresponding to ~10^7^ cells per milliliter. All fungi were fermented in PDB for 7 days and diluted to 10^6^ conidia per milliliter using a hemocytometer. Five treatment groups were established: (i) Pathogenic Fungi alone (PaF); (ii) Beneficial Fungi plus Pathogenic Fungi (BenF+PaF); (iii) Bacterial community plus Pathogenic Fungi (BacComm+PaF); (iv) Synthetic microbial community plus Pathogenic Fungi (SynMs+PaF); (v) sterile soil as a control (Control) (Table S7). The biomass ratio of the bacterial community was 1:1, and the biomass ratio of bacteria to fungi in the synthetic microbial community was 4:1. The final concentration of the synthetic microbial community in the soil was adjusted to 10^7^ cells per gram of soil. Each group of experiments consisted of nine independent biological replicates. The experiments were conducted under a 16-/8-h day/night cycle in an artificial incubator. The physiological indicators of the apple saplings were assessed as described above, and any mitigating effects of the synthetic microbial community on the pathogenic fungi was evaluated based on these physiological indicators.

To evaluate the preventive effects of the synthetic microbial community in replant soil mixtures from different production regions, three treatments were set for each region: (i) planting saplings in FS; (ii) inoculating sapling roots with the synthetic microbial community exhibiting optimal control efficacy followed by planting in replant soil (SynMs+RS); and (iii) direct replanting saplings in untreated orchard soil (RS). After 30 days of cultivation, apple growth was evaluated by measuring the plant height, fresh weights, total root surface, and root tip activity. The significant difference among the groups was determined by Kruskal–Wallis test with Dunn’s *post hoc* test (*P* < 0.05). The copy numbers of each pathogenic fungi were quantified with qPCR [46]. A standard curve was generated using serial 10-fold dilutions of a plasmid containing the fungal target gene. The threshold cycle values obtained for each sample were compared to the standard curve to calculate the copy number of the target gene. The amplification efficiency, determined from the slope of the standard curve, ranged from 96% to 100%. The specific primers targeted to each pathogenic fungi and PCR conditions are provided in Table S8. qPCR was performed using a Bio-Rad CFX96 Real-Time PCR system with SYBR qPCR Mixture (Vazyme Bio).

## Supplementary Material

Web_Material_uhaf217

## Data Availability

All data needed to support the conclusions in this paper are present in the paper and/or Supplementary data.
